# Rationale and design of the multidisciplinary team IntervenTion in cArdio-oNcology study (TITAN)

**DOI:** 10.1186/s12885-016-2761-8

**Published:** 2016-09-15

**Authors:** Edith Pituskin, Mark Haykowsky, Margaret McNeely, John Mackey, Neil Chua, Ian Paterson

**Affiliations:** 1 University of Alberta, Edmonton, AB Canada; 2 University of Texas, Arlington, TX USA; 3 Cross Cancer Institute, Edmonton, Alberta Canada; 4 Mazankowski Alberta Heart Institute, Edmonton, Alberta Canada; 5 4-256 Edmonton Clinic Health Academy, University of Alberta, Edmonton, T6G 1C9 AB Canada

**Keywords:** Multidisciplinary, Heart failure, Cardiac dysfunction, Lymphoma, Breast cancer

## Abstract

**Background:**

Cancer is the leading cause of premature death in Canada. In the last decade, important gains in cancer survival have been achieved by advances in adjuvant treatment. However, many oncologic treatments also result in cardiovascular "toxicity". Furthermore, cardiac risk factors such as hypertension, dyslipidemia, and diabetes mellitus are known to contribute to the progression of cardiac damage and clinical cardiotoxicity. As such, for many survivors, the risk of death from cardiac disease exceeds that of recurrent cancer. While provision of care by multidisciplinary teams has been shown to reduce mortality and hospitalizations among heart failure patients, the effect of assessments and interventions by multidisciplinary specialists in cancer patients receiving cardiotoxic chemotherapy regimens is currently unknown. Accordingly, we will examine the effect of a multi-disciplinary team interventions in the early assessment, identification and treatment of cardiovascular risk factors in cancer patients receiving adjuvant systemic therapy. Our main hypothesis is to determine if the incidence of LV dysfunction in cancer patients undergoing adjuvant therapy can be reduced through a multidisciplinary team approach.

**Methods/design:**

This is a randomized study comparing intensive multidisciplinary team intervention to usual care in the prevention of LV remodeling in patients receiving anthracycline or trastuzumab-based chemotherapy. Main objectives include early detection strategies for cardiotoxicity using novel biomarkers that reflect myocardial injury, remodeling and/or dysfunction; early identification and intensive treatment of cardiovascular risk factors; and early intervention with supportive care strategies including nutritional and pharmacist counselling, exercise training and cardiology team support. Secondary objectives include correlation of novel biomarkers to clinical outcomes; correlation of multidisciplinary interventions to adverse clinical outcomes; relationship of multidisciplinary interventions and chemotherapy dose density; preservation of lean muscle mass; and patient reported outcomes (symptom intensity and quality of life).

**Discussion:**

Cardiac toxicity as a result of cancer therapies is now recognized as a significant health problem of increasing prevalence. To our knowledge, TITAN will be the first randomized trial examining the utility of multidisciplinary team care in the prevention of cardiotoxicity. We expect our results to inform comprehensive and holistic care for patients at risk for negative cancer therapy mediated sequelae.

**Trial registration:**

ClinicalTrials.gov, NCT01621659 Registration Date 4 June 2012.

## Background

As a result of improved anti-cancer therapies, many patients now experience long-term survival after treatment. However, cardiac toxicity of cancer therapy is increasingly recognized as a major risk, such that for many survivors, the risk of death from cardiac disease exceeds that of recurrent cancer [1]. Negative lifestyle behaviors not only account for at least of 30 % of cancer deaths, but are well established causative factors for cardiovascular disease. Overweight/obesity, low fruit and vegetable intake coupled with high fat diet, physical inactivity, smoking and alcohol use are firmly established cancer-promoting, modifiable behaviors [2]. On this background of significant baseline cardiovascular risk, the ‘multiple-hits’ to the cardiovascular system with adjuvant chemotherapy convey varying degrees of directly negative effects [3]. Therefore, there is urgent need for effective interventions in this population.

The most common manifestation of cardiotoxicity during or after cancer therapy is left ventricular (LV) dysfunction and heart failure (HF) [4]. Cancer therapy-associated toxicity has been proposed to occur acutely (during infusion), early (within the first year of therapy) and chronic (>1 year post-therapy) [4]. Limat observed early and frequent cardiotoxicity in 135 consecutive lymphoma patients treated with CHOP (cyclophosphamide, doxorubicin, vincristine prednisone) [5]. Twenty-seven (20 %) patients suffered from a cardiac event within 1 year of treatment; among these, 14 patients had clinical signs of HF attributable to anti-cancer therapy. Long-term follow-up of patients after anthracycline-based therapies demonstrated abnormal cardiac function present in 18 % of patients followed up for less than 10 years and in 38 % of those followed for 10 years or more (median, 12 years) [6]. In 141 lymphoma patients assessed at least 5 years post-chemotherapy, Hequet observed subclinical cardiomyopathy in 39 (27.6 %) using echocardiography; associated risk factors included male gender, older age, and overweight [7]. Only 8 of these 39 patients received a doxorubicin dose > 300 mg/m^2^, indicating that even conservative doxorubicin dosing conveys long-term cardiac sequelae. These observations herald an emerging and potentially devastating health issue, as 60,000 patients per year are exposed to anthracyclines in the United States alone [8]. Maximizing the benefits and minimizing negative cardiac effects of anthracycline-based therapy is challenging. If cardiotoxicity is observed then anti-cancer treatment may be decreased or discontinued, potentially impacting remission rates [3]. Therefore, new approaches in early prevention and treatment of anthracycline-induced cardiotoxicity are required.

Our group has observed high prevalence and negative influence of cardiovascular risk factors in breast cancer patients. In 41 breast cancer patients who had received trastuzumab-based chemotherapy, we showed that at ~20 months following completion of therapy survivors consistently had higher rates of cardiovascular risk factors. Here, higher rates of overweight/obesity, low cardiorespiratory fitness (VO2_*peak*_) and unfavorable lipid profiles were observed compared to controls [9]. In 47 women who had received chemotherapy and endocrine therapy for hormone-receptor positive breast cancer, we again found consistently less favorable cardiovascular risk factors compared to controls. Higher resting heart rate and systolic blood pressure were observed; peak exercise output, stroke volume and cardiac power output were significantly lower in patients than controls [10]. While the cross-sectional designs limit our understanding of baseline characteristics, these results consistently identify significant clinical and sub-clinical negative cardiovascular effects of anti-cancer therapy. The major modifiable risk factors for cardiovascular disease (CVD) are well-established, and include tobacco use, high blood pressure (BP), high cholesterol, alcohol use, obesity and physical inactivity. Unfortunately, in oncology trials these risk factors have, to date, not been systematically examined. Retrospective reviews indicate modifiable cardiovascular risk factors including hypertension and coronary artery disease predict development of anthracycline-related cardiomyopathy [11, 12]. In patients receiving trastuzumab in the adjuvant setting, hypertension and high body mass index are associated with cardiac dysfunction [13, 14]. Taken together, research examining early identification of modifiable cardiovascular risk factors and intensive management to prevent negative sequelae is urgently required.

Importantly, LV dysfunction is now recognized as a late effect of chemotherapy, not detectable until significant damage has already occurred [15]. Unfortunately, when cardiotoxicity is detected, cardiology services are consulted late, if at all, and improvement in clinical cardiac outcomes is not always possible. In women receiving anthracycline-based chemotherapy, Cardinale showed a four-fold decrease in the chance of complete recovery from cardiac dysfunction for each doubling in time-to-heart failure treatment [16], emphasizing the urgency for early monitoring and intervention. Of note, the cardinal symptoms of HF (dyspnea, fatigue and edema) are common in cancer patients [17, 18], with 70–100 % reporting exercise intolerance and fatigue [19], and are difficult for the practitioner to distinguish from cardiac causes. Additionally, regular symptom inquiry [20, 21], routine vital sign measurements, and identification or management of cardiac risk factors [22, 23] are not routinely attended to in the oncology outpatient clinic. Taken together, these findings emphasize the importance of careful screening, early identification and intensive management of toxicities to ensure both adequate anti-cancer treatment is delivered on schedule and full dose, and that acute/chronic cardiovascular morbidity is prevented.

Patients at risk for or with cancer treatment-related cardiac effects are complex, multi-disease patients requiring multiple specialists [24], therefore, coordination of care by both cardiology and oncology specialties is urgently required. However, multiple challenges face clinicians managing this patient population including lack of: risk-stratification guidelines, distinguishing early toxicities, evidence-based cardiovascular monitoring schedules, and level 1 evidence for interventions. Furthermore, validation of new imaging techniques and improved biomarkers are necessary to individualize and monitor treatment.

### Our main hypothesis is to determine if the incidence of LV dysfunction in cancer patients undergoing adjuvant therapy can be reduced through a multidisciplinary team (MDT) approach

The main objectives include implementation of early detection strategies for cardiotoxicity with sensitive imaging modalities; early identification and intensive treatment of cardiovascular risk factors; and early intervention with supportive care strategies including nutritional counselling, pharmacist support, exercise training and cardiology team support. Secondary objectives include correlation of novel biomarkers to clinical outcomes; correlation of MDT interventions to adverse clinical outcomes; relationship of MDT interventions and chemotherapy dose density; preservation of lean muscle mass; and patient reported outcomes (symptom intensity and quality of life).

## Methods/design

TITAN (multidisciplinary Team IntervenTion in cArdio-oNcology) is a randomized study comparing intensive multidisciplinary team intervention to usual care in the prevention of LV remodeling in patients receiving anthracycline or trastuzumab-based chemotherapy. Additional inclusion requirements are shown in Table 1.

**Table 1:** Inclusion and Exclusion Criteria

Inclusion criteria	Exclusion criteria
• Patients receiving anthracycline or trastuzumab-based chemotherapy. • Histologically confirmed malignancy; • Age > 18 years, and • No contraindication to MRI.	• Physical disability preventing exercise testing or DEXA scan; • Psychiatric disease or disorder precluding informed consent; • Contraindication to anti-cancer therapy, including known heart failure, cardiomyopathy, or baseline LV ejection fraction less than 50 %; • Previous anthracycline or trastuzumab-based therapy; • Previous radiotherapy to thorax.

### Methods

The TITAN study underwent full Board review and was approved by the University of Alberta Health Research Ethics Board—Biomedical Panel. Potential participants are consecutively identified at Tumor Board review of all newly diagnosed cases of early breast cancer and lymphoma scheduled for chemotherapy (anthracycline and/or trastuzumab-based). Following primary oncologist approval, a study coordinator provides potential participants with an overview of the study and written information. Written informed consent to participate in the TITAN study is obtained from all participants. After informed consent, participants are scheduled for a baseline clinic visit for final determination of eligibility.

Clinical assessments include physical examination, cardiovascular history, symptomatology and risk factor profile. Smoking status, vital signs, menstrual status, weight and height will acquired. Cardiac MRI, coronary artery calcium score (cardiac CT) and body composition (Dual Energy X-ray Absorptiometry, or DEXA) will be acquired. Maximal aerobic capacity (peak pulmonary oxygen uptake or *VO*_*2peak*)_ is evaluated with an incremental exercise test; continuous expired gas analysis is performed with a metabolic measurement system. Heart rate and cuff blood pressure are measured at each stage; the highest oxygen consumed over 1-min will be used as the peak VO2 score [25, 26]. Upper and lower extremity maximal strength testing is also assessed. Detailed range of motion assessments are performed by an expert physiotherapist. In addition to clinically indicated laboratory, brain natriuretic protein, high-sensitivity troponin and research biofluids (collagen remodeling products, urine) are collected. Fasting lipid profile will be collected at baseline. Total energy expenditure and activity energy expenditure are evaluated with a SenseWear armband, worn by the participant for 4 days [27]. Quality of life will be assessed with instruments validated in cancer populations (‘Screening for Distress’ [28] and MDASI-HF [29], both based on the Edmonton Symptom Assessment System [30]). Each assessment will occur at baseline, 6 and 12 months. Our primary outcome is measure of left ventricular ejection fraction at 12 months.

### After baseline assessments, participants will be randomized 1:1 to multidisciplinary intervention or usual care (Fig. 1)

**Fig. 1:**
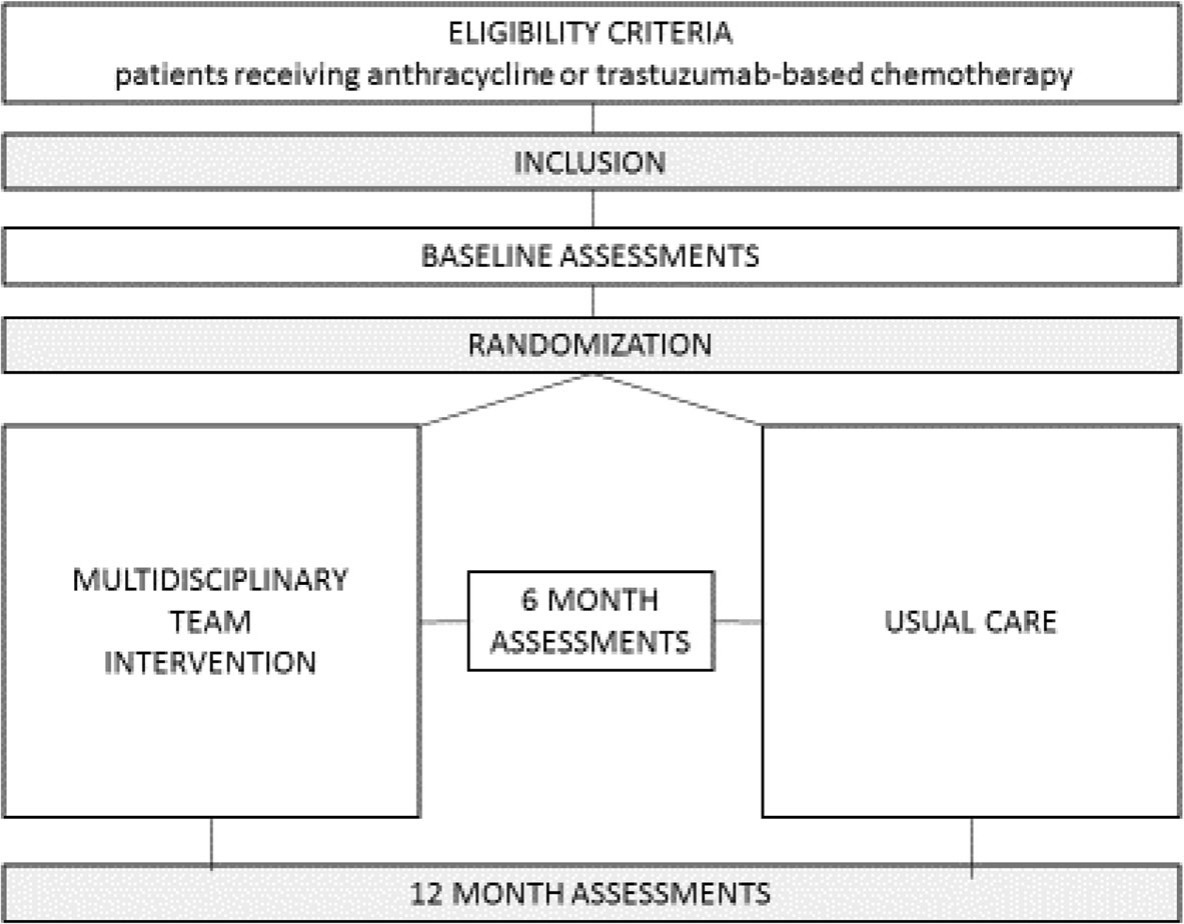
Flow chart TITAN Study

- Group 1: Multidisciplinary Team Intervention
  Patients randomized to the MDT arm will be assessed by the cardio-oncology team, and receive regular clinical assessment. Blood pressure and lipid profile targets will be guided by national guideline consensus documents [31, 32]. Counselling and written information regarding optimal nutrition will be provided by a registered dietician. As indicated, a clinical pharmacist will offer ongoing monitoring and support of any prescribed medications. Patients will be encouraged to participate in a supervised exercise training program consisting of endurance and resistance training twice per week and unsupervised endurance exercise (walking and/or cycling) exercise training another 1–2 days per week. During the first 3–4 months, moderate-intensity continuous aerobic training (30 to 60 min at 60 to 80 % peak VO_2_) will be performed on a cycle ergometer or treadmill. Thereafter, participants will perform moderate intensity contimuous exercise (1 day/week) and high-intensity aerobic interval training. Participants will also perform 1–3 sets of moderate upper (bench press, lattissimus dorsi puldown, shoulder press, arm curl and tricep extension) to lower extremity (leg press, leg extension and leg curl) resistance training (10 to 15 repetitons with weight added after 3 sets are completed while adhering to strict technique). All subjects will be given an exercise diary to record exercises performed, training heart rate, weight lifted and rate of perceived exertion.
- Group 2: Usual Care
  Patients randomized to usual care will receive standard anti-cancer therapy and standard clinical monitoring.

### Blinding and masking

The exercise assessments (baseline, 6 months and 1 year) are performed at a different facility by different staff than the supervised exercise training team. The cardiac MRI interpreter is blinded to the group assignment.

### Sample size calculations

The incidence of left ventricular systolic dysfunction in early breast cancer patients receiving trastuzumab has been reported as approximately 10 % in randomized clinical trials [33] and up to 22 % in non-trial community based practice [34]. The 1 year incidence of left ventricular dysfunction of early breast cancer patients receiving anthracyclines is not well characterized. In a randomized, placebo controlled trial on the prevention of cardiotoxicity of cancer patients receiving high dose anthracyclines, the incidence of LV dysfunction at 1 year was 43 % in the control arm vs. 0 % in the treatment arm [35]. Extrapolating from these results, we estimate that the 1 year incidence of left ventricular systolic dysfunction in our combined cohort of patients receiving trastuzumab and/or anthracyclines will be 15 % in those patients receiving usual care. Patients will be analyzed on an intent-to-treat basis. For patients attending the multidisciplinary clinic and receiving early pharmacologic intervention, we estimate the 1 year incidence of LV systolic dysfunction at 5 %. Given the high accuracy and reproducibility of cardiac MRI [36], with a two tailed significance level ∞ = 0.05 and power = 0.80 then 36 participants are required in each group. We anticipate a 10 % drop-out rate and a 3 % mortality during the 1 year follow-up so aim to recruit 40 patients in each arm.

### Data management

Study data are collected and managed using REDCap electronic data capture tools hosted at the University of Alberta [37]. REDCap (Research Electronic Data Capture) is a secure, web-based application designed to support data capture for research studies, providing 1) an intuitive interface for validated data entry; 2) audit trails for tracking data manipulation and export procedures; 3) automated export procedures for seamless data downloads to common statistical packages; and 4) procedures for importing data from external sources. Research biospecimens are stored within the Alberta Cancer Research Biobank [38] and maintained according to established standard operating procedures.

### Safety

Maximal exercise testing is safe for patients with early and metastatic cancer; the exercise protocol in this study has been used in healthy older subjects, cardiac patients and breast cancer survivors [10, 25, 26]. Participants experience normal effects of maximal exercise testing including sore muscles and temporary post-exercise fatigue.

## Discussion

Cardiac toxicity as a result of cancer therapies is now recognized as a significant health problem of increasing prevalence. Not only deleterious cardiac effects, but negative impacts on the entire oxygen cascade and multiple organ systems highlight the need for multiple interventional approaches as we will undertake here. To our knowledge, TITAN is the first randomized trial examining the effect of multidisciplinary team interventions in the prevention of chemotherapy-related cardiotoxicity. We expect that, in the short-term, this study will find that early multidisciplinary care will maintain cardiac geometry and enhance patient-reported quality of life. In the long-term, we anticipate that early supportive care interventions will prevent deleterious cardiovascular effects of cancer treatment, and improve overall survival (both cancer and cardiovascular). We also expect this work to inform new approaches in the delivery of comprehensive and holistic care in patients at risk for cancer therapy-mediated cardiovascular toxicity.

## Conclusion

Patients at risk for or with cancer treatment-related cardiac effects are complex, multi-disease patients requiring multiple specialists. The TITAN Study will provide high-level evidence in the development of guidelines for multidisciplinary preventive initiatives, locally, nationally and internationally. Lastly, this work will inform and support other long-term goals of our cardio-oncology team, in the prevention, detection and treatment of cardiovascular effects of cancer therapies.

## Abbreviations

CHOP: Cyclophosphamide, doxorubicin, vincristine prednisone
CT: Computerized tomography
DEXA: Dual energy X-ray absorptiometry
HF: Heart failure
LV: Left ventricle
MDT: Multidisciplinary team
MRI: Magnetic resonance imaging
VO2_*peak*_: Peak pulmonary oxygen uptake
